# Social capital in relation to alcohol consumption, smoking, and illicit drug use among adolescents: a cross-sectional study in Sweden

**DOI:** 10.1186/1475-9276-12-33

**Published:** 2013-05-20

**Authors:** Cecilia Åslund, Kent W Nilsson

**Affiliations:** 1 Centre for Clinical Research, Uppsala University, Västmanland County Hospital Västerås, Västerås, S-72189, Sweden

**Keywords:** Adolescence, Alcohol consumption, General social trust, Illicit drug use, Neighbourhood social capital, Smoking

## Abstract

**Background:**

Social capital has lately received much attention in public health research. However, few studies have examined the influence of social capital on alcohol consumption, smoking and drug use which have strong influence on public health. The present cross-sectional study investigated whether two measures of social capital were related to substance use in a large population of Swedish adolescents.

**Methods:**

A total of 7757 13–18 year old students (participation rate: 78.2%) anonymously completed the Survey of Adolescent Life in Vestmanland 2008 which included questions on sociodemographic background, neighbourhood social capital, general social trust, alcohol consumption, smoking, and illicit drug use.

**Results:**

Individuals within the group with low neighbourhood social capital had an approximately 60% increased odds of high alcohol consumption, more than three times increased odds of smoking and more than double the odds of having used illicit drugs compared with individuals with high neighbourhood social capital. Individuals within the group with low general social trust had approximately 50% increased odds of high alcohol consumption and double the odds of smoking and having used illicit drugs compared with individuals with high general social trust. However, social capital at the contextual level showed very weak effects on alcohol consumption, smoking, and illicit drug use.

**Conclusions:**

Social capital may be an important factor in the future development of prevention programs concerning adolescent substance use. However, further replications of the results as well as identifications of direction of causality are needed.

## Background

The concept of social capital has received much attention in the research field of public health. Although the concept has been much debated, social capital is still regarded as an important factor in understanding disparities in health. Social capital is related to a variety of health behaviours and outcomes including mental ill health [1-3], physical health [4-7], mortality [8,9], and violent crime [9,10]. Alcohol consumption, smoking, and drug use are well known risk factors in relation to public health [11-13]. Such health risk behaviours are often established during adolescence and may influence both the short and long term health of young people, where social capital may be an important factor.

Most research on social capital refers to the original definitions of the concept made by Bourdieu [14], Coleman [15], and Putnam [16,17], following the social school of Emile Durkheim [18]. The social capital concept includes a structural and a cognitive component that represent the norms and networks that enable people to collective action, co-operation and social participation [19]. The structural component includes societal aspects such as networks, connections and civic participation, whereas the cognitive component includes aspects of trust between individuals, social cohesion, and perceived social support [19]. Szreter and Woolcock [20] described three different forms of social capital: bonding, bridging and linking. Bonding social capital refers to: “trusting and co-operative relations between members of a network who see themselves as being similar, in terms of their shared social identity” [20]. Bridging social capital refers to: “relations of respect and mutuality between people who know that they are not alike in some socio-demographic (or social identity) sense (differing by age, ethnic group, class, etc.)” [20]. Linking social capital refers to: “norms of respect and networks of trusting relationships between people who are interacting across explicit, formal or institutionalized power or authority gradients in society” [20].

Social capital further has two important components related to the development of the concept within groups. The first component regards collectively shared norms, where a social or cultural norm is accepted and reinforced within a system, whether that system refers to a family, neighbourhood, or entire society. The second component regards social connectedness within the social structure [15,21]. The network density decides the collective power to influence and shape desirable behaviours among the individuals within the network.

As substance use is closely connected to societal norms it is not surprising that several studies on adult populations have found associations between social capital and alcohol consumption [22-24], smoking [22,25], and illicit drug use [26-28]. However, the measures of social capital differed widely between these studies, ranging from institutional and political trust [24,27], social participation [22,23,26,28], social trust [22,23,26-28], and community social trust [25].

Among US college students, associations have been found between social capital in the form of individual and community volunteerism in relation to alcohol use and harmful drinking outcomes [29-31]. Studies of US high school students have found associations between family social capital and use of alcohol, tobacco or drugs [32], as well as associations between secular trust and civic participation in relation to drug use [33]. Several other US studies have found associations between neighbourhood disorder and low social cohesion in relation to adolescent alcohol and drug use [34-36]. A study in England found that low neighbourhood sense of belonging was associated with smoking and low school sense of belonging was associated with drinking among adolescents [37]. In Japan, individual-level social trust at school was negatively associated with adolescent smoking and drinking behaviour [38]. In Sweden, negative correlations between social participation and trust in relation to smoking and illicit drug use, but not binge drinking, have been found among adolescents [39].

Several different aspects of social capital have been used in the previous studies as well as differences in findings which make the results difficult to generalize. There is thus a need for further investigations of the concept of social capital in relation to substance use in large, representative, adolescent populations.

There are societal norms and laws prohibiting adolescent drinking, smoking, and drug use in Sweden [40]. The social contexts and network closeness of the neighbourhood may thus, through the reinforcement of desired behaviours, influence adolescent substance use. This age group may be particularly affected by social capital of the neighbourhood because of limited mobility, i e the adolescent’s school, peer group, and part time activities are all confined to the neighbourhood [41]. Moreover, a low general social trust may result in different antisocial ways of coping with stress, peer evaluation, and conformity, which are common during adolescence [42,43] and could lead to substance use. The purpose of the present study is to investigate two components of social capital [44]: bonding social capital in the form of connectedness, networks and trust within neighbourhoods, and bridging social capital in the form of general social trust and feelings of connectedness with people in society in relation to alcohol consumption, smoking, and illicit drug use in a large population of Swedish adolescents. We hypothesized that low neighbourhood social capital and low general social trust would be associated with higher adolescent substance use.

## Methods

The present study was part of the Survey of Adolescent Life in Vestmanland 2008 (SALVe-2008), a survey distributed biannually by the County Council of Västmanland, Sweden. Västmanland is a medium-sized county consisting of ten municipalities, of which the largest municipality included more than half of the participants. All school students in 7th (13–14 years old) and 9th (15–16 years old) grade of elementary school, and 2nd (17–18 years old) grade of secondary school in the county, a total of 57 schools, were asked to complete a questionnaire during class hours. The questionnaire included questions about demographic background, neighbourhood social capital, general social trust, alcohol consumption according to AUDIT-C, smoking, and illicit drug use. Participation was anonymous and voluntary. The study followed the Swedish guidelines for studies of social science and humanities, according to the Declaration of Helsinki. According to Swedish regulations, this type of anonymous study no longer applies for ethical approval by the medical faculty.

A total of 7906 students completed the questionnaire, comprising 78.2% of the total population. The exclusion of 41 participants who did not state their sex and 108 participants, who did not complete the questionnaire or gave apparent incorrect answers, left 7757 participants for the analyses.

### Measures

*Sex*, whether the participant was a boy (0) or a girl (1).

*Parental unemployment*, whether both parents were working (0) or one or both parents were unemployed (1).

*Living conditions*, whether the participant was living in a single-family house (0) or a multi-family house (1).

*Ethnicity*, whether both parents were born in Sweden or Scandinavia (0) or at least one parent was born outside of Scandinavia (1).

#### Housing area SES

The participants stated their area of living out of 31 defined areas of the county. Statistical data of medium income of the general population for each area was obtained from Statistics Sweden. The variable was used as an objective measure of socioeconomic status (SES) of housing areas. Individual medium income in the county was € 18.287 (SD = € 1992), and medium income of the different housing areas ranged between € 13.815 – 24.651. The number of study participants living in each of the 31 housing areas ranged from 64 to 666.

The variable *Contextual SES* was created by ranking the 31 housing areas according to descending medium income, where rank 1 equals to the housing area with the highest income, and rank 31 equals to the housing area with the lowest income.

#### Subjective SES

Participants were asked to rank the SES of their family on a 7-point Likert scale by the following question: “Imagine society as being like a ladder. At the bottom are those with the least money, and at the top are those with the most. If you think about how wealthy your own family is compared with the rest of society, where would you place your family on this scale?” [45,46].

#### Neighbourhood social capital

Participants were asked to respond to seven statements about their neighbourhood (a part of a city, town or village): 1, In my neighbourhood, no-one needs to feel afraid. 2, It is unsafe to be outside at night in my neighbourhood. 3, In my neighbourhood, people seldom help each other. 4, If anything at home should break or go missing, we can always get help from a neighbour. 5, Most people know each other in my neighbourhood. 6, There are seldom any fights or trouble in my neighbourhood. 7, I often see graffiti and damaged objects (park benches, bus stops, street lights) in my neighbourhood. Response alternatives were: Strongly agree (1), Agree to some extent (2), Disagree to some extent (3), Strongly disagree (4). The use of this measurement has previously been reported in Åslund et al. [45]. The internal consistency (Chronbach’s alpha) of the neighbourhood social capital questions was 0.71.

A summation index was created with a range of 7–28 points where items 2, 3, and 7 were reversed. The index was also divided by quartiles, where the 1st quartile counted as high neighbourhood social capital, the 2nd-3rd quartiles counted as medium neighbourhood social capital, and the 4th quartile counted as low neighbourhood social capital.

The neighbourhood social capital variable was furthermore aggregated to housing area by ranking the 31 housing areas according to the medium neighbourhood social capital of each housing area. Rank 1 equals to the housing area with the highest neighbourhood social capital, and rank 31 equals to the housing area with the lowest neighbourhood social capital. In the statistical analyses, this variable is referred to as *Contextual neighbourhood social capital*.

#### General social trust

The participants were asked to respond to six statements about how they felt about society in general: 1, Most people can be trusted. 2, You can never be too careful when meeting new people. 3, Most people try to be helpful. 4, Most people would try to use others if they had the opportunity. 5, Most people only care about themselves. 6, Most people are honest. Response alternatives were: Strongly agree (1), Agree to some extent (2), Disagree to some extent (3), Strongly disagree (4). The use of this measurement has previously been reported in Åslund et al. [45]. The internal consistency of the general social trust questions was α = 0.73. A summation index was created with a range of 6–24 points, where items 2, 4 and 5 were reversed. The index was also divided by quartiles, where the 1st quartile counted as high general social trust, the 2nd-3rd quartiles counted as medium general social trust, and the 4th quartile counted as low general social trust.

The general social trust variable was furthermore aggregated to housing area by ranking the 31 housing areas according to the medium general social trust of each housing area. Rank 1 equals to the housing area with the highest general social trust, and rank 31 equals to the housing area with the lowest general social trust. In the statistical analyses, this variable is referred to as *Contextual general social trust*.

#### Alcohol consumption

The participants answered the first three questions of the Alcohol Use Disorders Identification Test (AUDIT-C), to measure alcohol consumption [47]. Previous studies support the use of the AUDIT [48,49] and AUDIT-C [50] in samples of secondary school adolescents. The following questions were asked: 1, How often did you have a drink containing alcohol in the past 12 months? Response alternatives were: Never (0), every second month or more seldom (1), about once a month (2), 2 to 4 times a month (3), 2 to 3 times a week (4), 4 times a week or more often (5). 2, How many glasses (a can or a bottle of beer, a glass of wine, a wine cooler, or one cocktail or a shot (4 cl) of hard liquor (like scotch, gin, or vodka) do you drink at a typical time when you are drinking alcohol? Response alternatives were: Do not drink alcohol (0), 1–2 glasses (1), 3–4 glasses (2), 5–6 glasses (3), 7–9 glasses (4), 10 or more glasses (5). 3, How often do you drink six or more glasses on one occasion? Response alternatives were: Never (0), every second month or more seldom (1), about once a month (2), 2 to 4 times a month (3), 2 to 3 times a week (4), 4 times a week or more often (5). Due to the young population, we modified the scale [51] by adding answer alternatives (Q 1; about once an month, and Q 3; 2 to 4 times a month) in comparison with the original AUDIT-C scale. This modification was made to avoid too broad gaps between the answer alternatives, which is presumably more suitable for measuring adolescent alcohol consumption in comparison with the original scale that is suited for adults. Previous studies of similar populations have shown that the modified version of the AUDIT-C has a Cronbach’s alpha coefficient of 0.95 and a Spearman’s *r* correlation coefficient of 0.96 compared with the full AUDIT form, as well as a high correlation (*r* = 0.733) to alcohol related problem behaviour [51-53]. The modified AUDIT-C index was created by summarising the scores of the three items (0–15 points). In the present study, the internal consistency of the modified AUDIT-C questions was α = 0.91. Furthermore, we created a dichotomized variable where the highest quartile within sex [51] were used as cut off value for high alcohol consumption. The cut off value for boys were one scale point lower than in the Nilsson et al. [51] study because the medium age of the present population was lower. Thus, Q3 within sex: boys ≥7, girls ≥6 were defined as high alcohol consumers in the present study. This cut-off allowed the analyses of the study to be presented on the whole population, and not split by sex.

#### Smoking

The participants responded to the question “Do you smoke?”. The response alternatives were 1) No, I have never smoked, 2) No, I have quit smoking, 3) Yes, I smoke once in a while, 4) Yes, I smoke every day. A smoker was defined as an individual who reported smoking every day. Thus, a dummy variable was created classifying smokers as 1, and non-smokers as 0.

#### Illicit drug use

The participants responded to the questions “Have you ever used hashish/marijuana?” and “Have you ever used other drugs than hashish/marijuana?” The response alternatives were 1) Never, 2) 1 time, 3) 2–4 times, 4) 5–10 times, 5) 11–20 times, 6) 21–50 times, 7) More than 50 times. A dummy variable was created for the logistic regression analyses where individuals who had used any kind of illicit drug at least once were classified as illicit drug users (1), and all other individuals were classified as illicit drug non-users (0).

### Statistical analyses

For analyses of the within-indexes reliability of neighbourhood social capital, general social trust, and the modified version of the AUDIT-C, Chronbach’s alpha was used. For analysis of correlations between the ordinal scaled indexes of individual neighbourhood social capital, contextual neighbourhood social capital, individual general social trust, contextual general social trust, subjective SES, contextual SES, alcohol consumption, smoking, and illicit drug use we used Spearman’s rho. General linear models (GLM) were used to investigate the relation between the non-independent control variables of sex, grade, parental unemployment, living conditions, and ethnicity, in relation to alcohol consumption, smoking, and illicit drug use.

As neighbourhood social capital and general social trust could be affected by intra-class correlation cluster effects hierarchical logistic regression analyses taking clustering effects into account were performed for analyses of relations between neighbourhood social capital, general social trust, and the three measures of substance use: the modified version of the AUDIT-C, smoking, and illicit drug use. First, a null model was performed [54]. Thereafter, individual and two-level models were performed. In the final model, the hierarchical logistic regression analysis was controlled for sex, grade, subjective SES, living conditions, parental unemployment, and ethnicity. Wald’s method for estimating parameters was used in the hierarchical logistic regression analyses. All statistical analyses were performed using SPSS (Statistical Package for Social Sciences 20.0) for Windows.

## Results

There were 1351 boys and 1394 girls in 7th grade, 1291 boys and 1314 girls in 9th grade, and 1230 boys and 1177 girls in 2nd grade of secondary school. Boys reported a slightly higher neighbourhood social capital and general social trust than girls (Table 1). Regarding the demographic data, 25.2% of the participants lived in a multi-family house, 20.8% had at least one unemployed parent, and 17.0% were of non-Scandinavian ethnicity, with no significant sex differences (not shown in tables). Mean subjective SES was somewhat higher among boys (*M* = 4.43, *95% CI* = 4.40-4.47, *SD* = 1.06) than among girls (*M* = 4.30, *95% CI* = 4.27-4.33, *SD* = 0.96, *p* <0.001) (not shown in tables).

**Table 1:** **Means, Medians, *SD*s, *95% CI*s and quartiles for individual and contextual neighbourhood social capital and individual and contextual general social trust, split on boys and girls, and *p*-values for sex differences**

	**Boys**						**Girls**						*** P***
	***N***	**Mean**	**Median**	***SD***	***95% CI***	***Q1-Q3***	***N***	**Mean**	**Median**	***SD***	***95% CI***	***Q1-Q3***	
Individual neighbourhood social capital	3698	22.08	22.00	3.79	21.96-22.20	20-25	3748	21.80	22.00	3.75	21.68-21.92	19-25	<0.001
Contextual neighbourhood social capital	3844	16.51	16.00	9.39	16.21-16.80	8-25	3875	16.50	16.00	9.53	16.20-16.80	8-25	0.816
Individual general social trust	3628	15.22	15.00	3.01	15.12-15.32	13-17	3656	14.87	15.00	2.90	14.77-14.96	13-17	<0.001
Contextual general social trust	3844	15.77	14.00	9.20	15.48-16.01	9-23	3875	15.75	14.00	9.39	15.46-16.05	8-24	0.762

As shown in Table 2, the prevalence of high alcohol consumption, everyday smoking, and any illicit drug use increased with grade and were most common in the 2nd year of high school. Moreover, smoking was more common among girls and illicit drug use was more common among boys (Table 2). Moreover, the intra class correlation (ICC) of alcohol consumption, smoking, and illicit drug use was 0.346, p < 0.001.

**Table 2:** **Percentages of participants reporting high alcohol consumption, everyday smoking, and use of any illicit drug at least once, divided by school year and sex, with χ**^**2 **^**and *p*-values for sex differences**

	**7th grade**			**9th grade**			**2nd grade (secondary school)**		
	**Boys % ( *n *)**	**Girls % ( *n *)**	**χ**^**2 **^**( *p *)**	**Boys % ( *n *)**	**Girls % ( *n *)**	**χ**^**2 **^**( *p *)**	**Boys % ( *n *)**	**Girls % ( *n *)**	**χ**^**2 **^**( *p *)**
High alcohol consumption	3.7 (48)	8.1 (109)	22.62***	24.0 (298)	25.9 (333)	1.30 *ns*	51.5 (620)	47.5 (553)	3.76 *ns*
Everyday smoking	2.1 (28)	3.1 (43)	2.54 *ns*	8.0 (100)	9.6 (124)	2.07 *ns*	9.1 (110)	15.4 (181)	22.33***
Used any illicit drug at least once	4.5 (58)	4.7 (63)	0.021 *ns*	12.3 (153)	8.7 (112)	8.61**	22.7 (271)	17.4 (201)	10.25***

* p < = 0.05, ** p < = 0.01, *** p < = 0.001.

Higher scores of neighbourhood social capital and general social trust were correlated with lower alcohol consumption, less smoking, and less illicit drug use (Table 3). The variables of neighbourhood social capital and general social trust were moreover correlated with both subjective SES and contextual SES (Table 3). The contextual neighbourhood social capital and contextual general social trust showed non-significant or very weak correlations with alcohol consumption, smoking, and illicit drug use (Table 3). The ICC of the contextual level predictor variables was -0.011, *p* = 0.945, and the ICC of the individual level predictor variables was 0.260, *p* <0.001.

**Table 3:** Correlations with Spearman’s rho

**Factors**^**a**^	**1.**	**2.**	**3.**	**4.**	**5.**	**6.**	**7.**	**8.**	**9.**	**10.**
1. Individual neighbourhood social capital	1	-0.286***	0.302***	-0.210***	0.183***	0.204***	-0.109***	-0.161***	-0.155***	-0.126***
2. Contextual neighbourhood social capital	-	1	-0.75***	0.644***	0.000 *ns*	-0.488***	-0.17 *ns*	0.022 *ns*	0.025*	-0.004 *ns*
3. Individual general social trust	-	-	1	-0.123***	0.126***	0.055***	-0.193***	-0.188***	-0.155***	-0.119***
4. Contextual general social trust	-	-	-	1	-0.006 *ns*	-0.404***	0.020 *ns*	0.037 **	0.051***	0.013 *ns*
5. Subjective SES	-		-		1	0.059***	-0.046***	-0.105***	-0.055***	-0.037
6. Contextual SES	-		-		-	1	0.027*	-0.010	-0.035**	-0.025*
7. Alcohol consumption	-		-		-	-	1	0.589***	0.385***	0.259***
8. Smoking	-		-		-	-	-	1	0.434***	0.323***
9. Use of hashish/marijuana	-		-		-	-	-	-	1	0.597***
10. Use of other narcotics	-		-		-	-	-	-	-	1

* p < = 0.05, ** p < = 0.01, *** p < = 0.001.

In binary logistic regressions, the null model (without any explanatory variables) for alcohol showed OR = 0.345 (*95% CI* = 0.326-0.364, *p* <0.001). The null model for smoking showed OR = 0.087 (*95% CI* = 0.079-0.095, *p* <0.001), and the null model for illicit drug use showed OR = 0.133 *(95% CI* = 0.123-0.143, *p* <0.001). The unadjusted logistic regression models revealed significant effects of having low neighbourhood social capital and low general social trust in relation to high alcohol consumption, smoking, and illicit drug use (Table 4, Model 1). Hierarchical logistic regression analyses were further performed to control for intra-class correlation clustering effects of housing area (Table 4, Models 2, 3, and 4). There were however very similar estimates in the unadjusted logistic regression models and the two-level models (individuals clustered within housing area). The ICC of the two-level predictor variables was -0.001, *p* = 0.349.

**Table 4:** Multilevel analysis

	**High alcohol consumption**		**Smoking**		**Illicit drug use**	
**Model 1, Individual level unadjusted logistic regression**						
	***OR (95% CI)***	***p***	***OR (95% CI)***	***p***	***OR (95% CI)***	***p***
*Individual neighbourhood social capital*						
High	Ref		Ref		Ref	
Medium	0.97 (0.83-1.13)	*ns*	1.68 (1.22-2.32)	0.001	1.36 (1.06-1.73)	0.016
Low	1.34 (1.13-1.58)	0.001	3.37 (2.43-4.69)	<0.001	2.67 (2.06-3.44)	<0.001
*Individual general social trust*						
High	Ref		Ref		Ref	
Medium	1.62 (1.37-1.92)	<0.001	1.53 (1.11-2.12)	0.010	1.52 (1.16-1.98)	0.002
Low	2.26 (1.89-2.71)	<0.001	2.66 (1.90-3.71)	<0.001	2.81 (2.14-3.70)	<0.001
**Model 2, Two-level hierarchical logistic regression analyses with contextual level SES**						
	***OR (95% CI)***	***p***	***OR (95% CI)***	***p***	***OR (95% CI)***	***p***
Contextual SES	1.01 (1.01-1.02)	<0.001	1.00 (0.99-1.01)	*ns*	1.00 (0.99-1.01)	*ns*
*Individual neighbourhood social capital*						
High	Ref		Ref		Ref	
Medium	1.00 (0.85-1.17)	*ns*	1.78 (1.26-2.52)	0.001	1.29 (1.00-1.67)	*ns*
Low	1.48 (1.24-1.77)	<0.001	3.63 (2.53-5.20)	<0.001	2.66 (2.03-3.48)	<0.001
*Individual general social trust*						
High	Ref		Ref		Ref	
Medium	1.65 (1.39-1.97)	<0.001	1.65 (1.17-2.34)	0.004	1.50 (1.14-1.98)	0.004
Low	2.29 (1.89-2.77)	<0.001	2.76 (1.93-3.94)	<0.001	2.76 (2.08-3.67)	<0.001
**Model 3, Two-level hierarchical logistic regression analyses with contextual level neighbourhood social capital**						
	***OR (95% CI)***	***p***	***OR (95% CI)***	***p***	***OR (95% CI)***	***p***
Contextual neighbourhood social capital	0.99 (0.98-0.99)	<0.001	1.00 (0.99-1.01)	*ns*	0.99 (0.99-1.00)	*ns*
*Individual neighbourhood social capital*						
High	Ref		Ref		Ref	
Medium	1.001 (0.87-1.17)	*ns*	1.71 (1.23-2.36)	0.001	1.37 (1.07-1.76)	0.012
Low	1.47 (1.24-1.76)	<0.001	3.44 (2.45-4.83)	<0.001	2.77 (2.13-3.61)	<0.001
*Individual general social trust*						
High	Ref		Ref		Ref	
Medium	1.65 (1.39-1.95)	<0.001	1.51 (1.09-2.10)	0.013	1.51 (1.16-1.97)	0.002
Low	2.30 (1.92-2.76)	<0.001	2.63 (1.88-3.67)	<0.001	2.81 (2.14-3.69)	<0.001
**Model 4, Two-level hierarchical logistic regression analyses with contextual level general social trust**						
	***OR (95% CI)***	***p***	***OR (95% CI)***	***p***	***OR (95% CI)***	***p***
Contextual general social trust	1.00 (0.99-1.00)	*ns*	1.00 (0.99-1.01)	*ns*	1.01 (1.00-1.01)	*ns*
*Individual neighbourhood social capital*						
High	Ref		Ref		Ref	
Medium	0.97 (0.83-1.13)	*ns*	1.71 (1.24-2.37)	0.001	1.33 (1.04-1.71)	0.022
Low	1.36 (1.15-1.61)	<0.001	3.47 (2.48-4.85)	<0.001	2.59 (2.00-3.36)	<0.001
*Individual general social trust*						
High	Ref		Ref		Ref	
Medium	1.65 (1.39-1.95)	<0.001	1.52 (1.09-2.10)	0.012	1.50 (1.15-1.96)	0.003
Low	2.31 (1.93-2.77)	<0.001	2.64 (1.89-3.68)	<0.001	2.77 (2.11-3.65)	<0.001
**Model 5, Two-level hierarchical logistic regression analysis adjusted for confounding factors**						
	***OR (95% CI)***	***p***	***OR (95% CI)***	***p***	***OR (95% CI)***	***p***
Contextual SES	1.00 (0.99-1.01)	*ns*	1.00 (0.99-1.02)	*ns*	1.00 (0.99-1.01)	*ns*
Contextual neighbourhood social capital	1.00 (0.99-1.02)	*ns*	1.01 (1.00-1.03)	*ns*	0.98 (0.97-1.00)	0.011
Contextual general social trust	0.98 (0.97-0.99)	0.005	0.98 (0.97-1.00)	*ns*	1.02 (1.00-1.03)	0.046
*Individual neighbourhood social capital*						
High	Ref		Ref		Ref	
Medium	1.07 (0.89-1.28)	*ns*	1.79 (1.24-2.59)	0.002	1.35 (1.03-1.77)	0.029
Low	1.57 (1.27-1.94)	<0.001	3.12 (2.11-4.60)	<0.001	2.55 (1.90-3.43)	<0.001
*Individual general social trust*						
High	Ref		Ref		Ref	
Medium	1.32 (1.09-1.60)	0.005	1.38 (0.96-1.97)	*ns*	1.29 (0.97-1.73)	*ns*
Low	1.50 (1.21-1.85)	<0.001	1.91 (1.32-2.77)	0.001	2.03 (1.50-2.74)	<0.001
Sex	1.05 (0.93-1.19)	*ns*	1.40 (1.15-1.71)	0.001	0.67 (0.56-0.97)	<0.001
Grade	3.54 (3.24-3.87)	<0.001	1.93 (1.69-2.20)	<0.001	2.17 (1.94-2.43)	<0.001
Parental unemployment	0.88 (0.75-1.04)	*ns*	1.34 (1.07-1.69)	0.012	1.14 (0.93-1.39)	*ns*
Living conditions	1.19 (1.02-1.40)	0.029	1.41 (1.12-1.76)	0.003	1.27 (1.04-1.54)	0.018
Ethnicity	0.62 (0.51-0.75)	<0.001	0.88 (0.68-1.15)	*ns*	1.04 (0.83-1.30)	*ns*
Subjective SES	0.93 (0.82-1.06)	*ns*	1.27 (1.05-1.55)	0.016	1.09 (0.92-1.28)	*ns*

Model 1 is the individual level unadjusted logistic regression. Models 2, 3, and 4 show the two-level hierarchical logistic regression analyses which consider non-independent effects of contextual level socioeconomic status (SES), contextual level neighbourhood social capital, and contextual level general social trust (all aggregated to housing area). Model 5 shows the two-level hierarchical logistic regression analysis which considers the individual level neighbourhood social capital, individual level general social trust, contextual level SES, contextual level neighbourhood social capital, and contextual level social trust, adjusted for confounding factors.

Further, the association between neighbourhood social capital and general social trust in relation to substance use could have been affected by non-independent covariates such as sex, grade, parental unemployment, living conditions, ethnicity, and subjective SES. In general linear models these non-independent covariates were all related to the independent (neighbourhood social capital and general social trust) and dependent (alcohol consumption, smoking, and illicit drug use) variables of the study (*p* <0.05), except for sex and parental unemployment in relation to alcohol consumption, and ethnicity in relation to smoking (data not shown). Therefore, the final hierarchical logistic regression model was further adjusted for the non-independent covariates of sex, grade, parental unemployment, living conditions, ethnicity, and subjective SES (Table 4, Model 5). Individuals within the group with low neighbourhood social capital had an odds ratio of 1.57 for high alcohol consumption, an odds ratio of 3.12 for smoking, and an odds ratio of 2.55 for illicit drug use compared with individuals with high neighbourhood social capital (Table 4, Model 5). Individuals within the group with low general social trust had an odds ratio of 1.50 for high alcohol consumption, an odds ratio of 1.91 for smoking, and an odds ratio of 2.03 for illicit drug use compared with individuals with high general social trust (Table 4, Model 5). The adjustment for the non-independent variables did somewhat alter the effects of neighbourhood social capital and general social trust in relation to alcohol consumption, smoking, and illicit drug use that were found in the unadjusted regression models. The differences in effects were mainly explained by the adjustment for grade, where higher grades were associated with increased odds of alcohol consumption, smoking, and illicit drug use.

## Discussion

The present study investigated whether bonding social capital in the form of connectedness and networks within neighbourhoods (neighbourhood social capital) and bridging social capital in the form of general social trust were associated with substance use in a large Swedish adolescent population. We found that both low neighbourhood social capital and low general social trust were associated with higher alcohol consumption and higher prevalence of smoking and illicit drug use. The results remained after adjusting for possible confounding from demographic and socioeconomic factors, thus demonstrating robust findings of the models.

Low neighbourhood social capital was associated with an approximately 60% increased odds of high alcohol consumption, more than three times increased odds of smoking and more than double the odds of having used illicit drugs. Low general social trust was associated with an approximately 50% increased odds of high alcohol consumption and double the odds of smoking and having used illicit drugs.

It seems plausible that low levels of bonding social capital within the neighbourhood might be associated with a lack of social control, lower community reinforcements of desired behaviours, and lower tendency to conform to the norms of society, including norms and laws prohibiting underage drinking, smoking, and drug use. Low bonding social capital of the neighbourhood may also be more prevalent in socioeconomically deprived areas, with higher prevalence of social problems, youth delinquency and availability of illegal drugs. Moreover, low levels of bridging social capital in the form of general social trust might result in a weaker identification with the society and the norms of society and less tendency to conform to these norms, including norms prohibiting underage drinking, smoking, and drug use. It may also involve feelings of insecurity and alienation which could lead to higher stress levels and antisocial coping behaviours including substance use.

The results are in accordance with previous findings in adult populations [22,23,25-27] as well as previous findings regarding alcohol consumption [34], smoking and illicit drug use [34,39] among adolescents. However, our finding of an increased risk of high alcohol consumption among adolescents with lower levels of general social trust differed from the previous finding by Lundborg [39]. This may be explained by differences in alcohol measures, the use of one community setting in the Lundborg study compared with the ten community/whole county setting of the present study, and the larger population sample of the present study (*n* > 7000). The weaker results found for alcohol consumption in relation to neighbourhood social capital in comparison with smoking and illicit drug use may also be explained by the fact that alcohol consumption was more frequently occurring in the adolescent population than smoking and illicit drug use. Moreover, it is possible that alcohol consumption may be more socially accepted than smoking and illicit drug use among Swedish adolescents considering the substantially higher proportion of alcohol users versus tobacco and illicit drug users [55]. This could involve less controlling and regulating effects of neighbourhood social capital on alcohol use. This possible explanation would be interesting to investigate in future studies.

At the contextual level, weak relations were found. In the adjusted two-level model, housing area social trust in relation to alcohol consumption showed decreased odds of 1–3%, whereas an increased odds of 1–3% was found in relation to illicit drug use. Contextual level neighbourhood social capital was related to illicit drug use with increased odds of 1–3%. In previous contextual level analyses of social trust within schools Takakura found a 30% increased odds for smoking among students in schools with lower levels of trust although the association was not statistically significant [38]. Our findings of contextual level estimates in relation to alcohol consumption, smoking, and illicit drug use were distinctly weaker. Our significant findings are probably a consequence of the larger population sample which elevated the power in the present analyses. However, an interpretation of these results might also be that contextual level effects and social processes within groups have smaller effects in Sweden than in Japan. Sweden is known for its highly egalitarian and individualistic society. Swedes have been suggested to have a strong need for social autonomy and to not be dependent on other individuals, such as neighbours, relatives, employers, etc. [56]. This could be an explanation for the weak contextual level cluster effects of housing area in the present study. Moreover, it is interesting that even in a highly egalitarian country such as Sweden there is an association between individual level neighbourhood social capital, general social trust and substance use in an adolescent population. Social capital has been suggested as one important mediating factor of the relations between income inequality and ill health [9,57-59] and substance use is strongly related to public health [11-13].

The results of the present study should be interpreted in the light of several limitations. Firstly, the debate regarding the concepts of social capital and social trust and how they are supposed to be measured is not settled. Social capital is operationalized in many different ways depending on which theoretical framework the researcher uses. The different schools of sociology often employ either a consensus or a conflict perspective, or a macro or micro perspective, which always attract criticism from the opposing side [60]. For example, Putnam [16,17] describes social capital by using a functionalist, consensual perspective, whereas Bourdieu [14] defines social capital as more based on conflict and exploitation. In the present study, we aimed to determine whether two forms of bonding and bridging social capital [44] were related to substance use among adolescents. Our measures are corresponding to Putnam’s description of social capital [16,17]. However, this way of using the concept of it can always be criticised depending on whether a consensus, conflict, macro, or micro perspective is used [60].

Secondly, the analyses are based mainly on self-reports, which involves a risk of information bias due to false or inaccurate responses from the participants. Individuals are often inclined to underreport behaviours that are not approved of in society and/or against cultural norms. Thus, the underreporting of alcohol consumption and illicit drug use are well-known problems within the research field of substance use and misuse when using self-reports [61-63]. The prevalence of adolescent substance use corresponded to previous national studies in Sweden [55]. However, the AUDIT-C has only been validated in the oldest age group of the present study [50]. The measures of smoking and illicit drug use were not validated. To our knowledge, there is no validated gold standard for the measurement of social capital. Regarding the validity of the social capital measures, we have considered the construct validity, face validity, and content validity of the measurements as suggested by Harpham et al. [44]. Moreover, this study design always involves a risk of confounding from unmeasured variables, for example parental education, peer attitudes to substance use, group pressure, etc. However, we have adjusted for several other well-known and previously suggested confounders in our models [44].

Thirdly, there is the problem of causality regarding neighbourhood social capital, general social trust and substance use, as the cross-sectional design of the study involved no possibility to distinguish the directions of cause and effect. Although the directions of causality implied in the models of the study, where low neighbourhood social capital and general social trust were associated with higher levels of substance use, seems plausible, substance use may also alter and influence levels of neighbourhood social capital and general social trust. The data moreover did not allow analyses of how an individual’s neighbourhood referred to in the neighbourhood social capital measurement was related to school and housing area.

Lastly, adolescents as a group may be particularly affected by social capital of the neighbourhood as a cause of limited mobility [41]. Limited mobility can also be related to the outcome, e g underage drinking if alcohol outlets are not dense in the residential area. However, in Sweden there are no legal ways for adolescents to obtain alcohol, due to the alcohol monopoly of the Swedish government not permitting individuals under the age of 20 to purchase alcohol. Therefore, limited mobility in regard to alcohol outlets would not be of any great influence in this study.

The study also has several strengths, particularly regarding the large adolescent community sample from a county that is considered to be fairly representative of Sweden as a whole, because of its distribution of educational, income, and employment levels as well as urban and rural areas [64]. The rather high participation rate reduces the risk of selection bias. The results may thus be possible to generalize to other adolescent populations.

## Conclusions

Our findings suggest a link between subjective neighbourhood social capital, general social trust, and substance use in a Swedish adolescent population. Given that our findings are valid, they contribute important information to the on-going debate on social capital and public health by demonstrating associations between two social capital factors and alcohol consumption, smoking, and illicit drug use among adolescents. Social capital may be an important factor in the future development of prevention programs concerning adolescent substance use. However, further replications of the results and identifications of direction of causality are needed before any interventions may be suggested.

## Competing interests

The authors declare that they have no competing interests.

## Authors’ contributions

Study concept and design: CÅ, KWN. Acquisition of data: CÅ, KWN. Questionnaire management: CÅ. Analysis and interpretation of data: CÅ, KWN. Drafting of manuscript: CÅ, KWN. Critical revision: CÅ, KWN. Both authors read and approved the final manuscript.
